# Growth and Lipidomic Responses of Juvenile Pacific White Shrimp *Litopenaeus vannamei* to Low Salinity

**DOI:** 10.3389/fphys.2019.01087

**Published:** 2019-08-23

**Authors:** Maoxian Huang, Yangfan Dong, Yan Zhang, Qinsheng Chen, Jia Xie, Chang Xu, Qun Zhao, Erchao Li

**Affiliations:** ^1^Key Laboratory of Tropical Biological Resources of Ministry of Education, Hainan University, Haikou, China; ^2^Department of Aquaculture, College of Marine Sciences, Hainan University, Haikou, China

**Keywords:** salinity, *Litopenaeus vannamei*, lipidomics, growth, gill, muscle

## Abstract

The Pacific white shrimp (*Litopenaeus vannamei*), a euryhaline penaeid species, can tolerate a wide range of salinities, but little is known on its strategies to cope with low salinity fluctuations from the aspect of lipidomics. Thus, in this study, *L. vannamei* were grown in two different salinities [3 and 30‰ (control)] for 8 weeks, and then an liquid chromatography (LC)–mass spectrometry (MS)-based lipidomics analysis was performed to reveal the lipid profile differences in gill and muscle. *L. vannamei* under low salinity had lower weight gain and condition factor than the control shrimp at 30‰, but no differences were found in survival and hepatopancreas index. A higher number of differential lipid metabolites were identified in gill than in muscle in *L. vannamei* at salinity 3‰ relative to the control shrimp at salinity of 30‰ (159 versus 37), which belonged to 11 and 6 lipids classes, respectively. Of these lipids, phosphatidylcholine (PC), phosphatidylinositol (PI), phosphatidic acid (PA), phosphatidylethanolamine (PE), and triglyceride (TG) were the main lipids in both shrimp gill and muscle, regardless of salinities. Compared with the control shrimp at salinity 30‰, the percentage of PC significantly reduced, but TG and PA significantly increased in gill of shrimp at salinity 3‰. Moreover, the relative fatty acid abundances showed significant changes in *L. vannamei* between the two salinity groups, but the patterns of the changes were complex and were fatty acid dependent. Neither lipid nor fatty acid composition in muscle was affected by salinity. Further pathway analysis showed that these metabolites were closely related to lipid and fatty acid metabolic pathways. All the findings in this study reveal that the lipid variations are closely related to bio-membrane structure, mitochondrial function, energy supply, or organic osmolyte contents in hemolymph for improving osmoregulatory capacity of *L. vannamei* under low salinity.

## Introduction

The Pacific white shrimp (*Litopenaeus vannamei*) is found in tropical waters, from Mexico to Peru, and in the last decade, there was an increase of using this species in inland aquaculture (Valencia-Castañeda et al., 2018). Regarded as a highly efficient osmoregulator, *L. vannamei* can tolerate a wide range of salinities from 0.5 to 50‰ (Saoud et al., 2003; Xu et al., 2018). Salinity is an important environmental factor, however, has been shown to influence the distribution, abundance, and physiological response of aquatic animals extensively (Lv et al., 2013; Penney et al., 2016). Poor survival and growth, as well as low stress tolerance and disease resistance, have become restrictive factors for inland low salinity (<5‰) *L. vannamei* farming (Lin and Chen, 2003; Li et al., 2007, 2008). To solve these problems osmoregulatory adjustments are necessary to control hemolymph osmotic pressure, as well as the development of compensatory mechanisms allowing re-establishment of homeostasis before cellular structure and function are severely disturbed (Brito et al., 2000; Lucu et al., 2008; Silva et al., 2010).

Aquatic animals require more energy from growth for osmoregulation at low salinity (Spaargaren, 1975; Li et al., 2008; Huong et al., 2010). A great amount of extra energy in *L. vannamei* (20–50% of total metabolic energy) are needed for osmoregulatory processes including increasing metabolic rate, modification of cellular membrane components, alterations in ion transport enzyme activity, highly unsaturated fatty acids (HUFAs) concentration, free amino acids (FAAs) concentration, water permeability of gills, and synthesis of certain non-essential amino acids under hyposaline stress (Evans et al., 2005; Hurtado et al., 2007; Lv et al., 2013; Chen et al., 2014; Penney et al., 2016; Faleiros et al., 2018). Thus, it is reasonable to explore the effect of energy provision on osmoregulatory efficiency of *L. vannamei* grown in a low-salinity environment.

Of the three energy-yielding nutrients, lipids may be closely related to osmoregulation, because lipids have the greatest energy density, and many fatty acids derived from lipid metabolism are essential for normal growth and various metabolic functions in shrimp (Li et al., 2017). Previous studies have shown that large amounts of neutral lipids could be stored in cells for membrane synthesis and energy supply in times of starvation (Li et al., 2007; Tseng and Hwang, 2008). Moreover, phospholipids and glycolipids are indispensable components of the cell membrane, effecting osmoregulatory capacity of crustaceans by changing their cell membrane structures (Chapelle and Zwingelstein, 1984; Li et al., 2006; Chen et al., 2014). Furthermore, increasing dietary lipid level from 6 to 9% can alleviate the osmoregulatory pressure of *L. vannamei* under hyposaline stress (Xu et al., 2018). However, information is limited on the physiological functions of specific lipids and fatty acid metabolites in improving osmoregulatory efficiency of shrimp under low salinity.

Lipidomics is a mass spectrometry-based science for exploring the structure, composition, and even quantity of lipids in biological systems such as cells, organs, and body fluids (Smith et al., 2014). Gills, as the organs primarily responsible for osmoregulation of hemolymph, specialize in exchange with the exterior medium, and muscle is the material repository or nutrient pool for many of the aquatic organisms (Tseng and Hwang, 2008). However, little is known on the lipidomic characteristics in either gill or muscle in *L. vannamei* under low salinity. Therefore, this study investigated the significant differences in lipid metabolites between shrimp grown in salinity of 3 and 30‰ via ultra performance liquid chromatography–mass spectrometry (UPLC–MS) analysis. The current study is first investigation examining the effect of hyposaline stress on *L. vannamei* using lipidomics analysis. The results in this study would help to elucidate the physiological strategies of *L. vannamei* for adapting to low salinity, and provide new insights into the significance of lipids for osmoregulation of *L. vannamei*.

## Materials and Methods

### Experimental Animals and Design

Healthy juvenile *L. vannamei* were obtained from a shrimp larvae breeding base in Danzhou, Hainan, China. Shrimp were acclimatized for 1 week at 30‰ salinity in three tanks. During acclimation, salinity of two tanks was adjusted to 3‰ by decrease water salinity 3–5‰ per day by aerated and de-chlorinated tap water. Then, a total of 160 juvenile *L. vannamei* (0.75 ± 0.03 g) were randomly divided into separated tanks with four replicates for each salinity group with 20 shrimps per tank. During the acclimation and experimental periods, shrimp were fed three times daily at 08:00, 16:00, and 22:00 with commercial feed for *L. vannamei*. Based on the amount of residual food, daily rations were adjusted to a feeding level slightly more than satiation. The uneaten food and excrement were removed with a siphon tube once daily with water renewal (50%) until the end of the tests. The photoperiod was 12 h light and 12 h dark. Water pH (7.5–7.9), temperature (26–28°C), dissolved oxygen (4.8–6.4 mg/L), and total ammonia nitrogen concentration (< 0.02 mg/L) were monitored twice a week and maintained throughout the experiment.

### Growth Evaluation and Sampling

After 8 weeks, all shrimp were fasted for 24 h prior to sampling. Shrimp in each tank were bulk weighed and counted. Then, each shrimp was dissected rapidly to obtain the hepatopancreas, gills, and muscle tissues on ice. Hepatopancreases were weighed to determine the hepatosomatic index. Gills and muscles from four individuals in each tank were pooled as one sample and frozen in liquid nitrogen immediately, and then were kept at −80°C for lipidomics analysis. Growth performance-related parameters were calculated as follows:

Survival (%) = (final number of prawns/initial number of prawns) × 100;
Weight gain (%) = ((final weight−initial weight)/initial weight) × 100;
Condition factor (%) = (final weight (g)/(body length (cm))^3^) × 100;
Hepatosomatic index (%) = (wet hepatopancreas weight (g)/wet body weight (g)) × 100.

### Lipid Extraction

The methods for lipid extraction were according to Bi et al. (2013) with modifications. Gill and muscle samples selected from each tank were excised (50 mg) and dissolved in 1.5 mL trichloromethane/methanol (2/1, v/v), then mixed with 0.5 mL of ultrapure water (Merck, Germany). After being vortexed for 1 min, the solutions were separated by centrifugation at 3000 rpm for 10 min. The total organic phase of each was separated and placed in a clean test tube and dried under N_2_, then re-dissolved in 400 μL isopropanol/methanol (1/1, v/v). After centrifugation at 3000 rpm for 10 min at 4°C, the upper phase was transferred for following analysis.

### LC–MS Analysis

Untargeted UPLC analysis was performed using the Ultimate 3000 LC system (Dionex, United States) with a Kinetex C18 analytical column (100 × 2.1 mm, 1.9 μm). The injection volume was 4 μL. The mobile phase was composed of solvents A (60% acetonitrile/40% H_2_O, v/v) and B (10% acetonitrile/90% isopropanol, v/v); the former contained 10 mmol/L ammonium formate and the latter contained 10 mmol/L ammonium formate and 0.1% formic acid. In addition, the concentration of solvent B underwent an optimized gradient program. It was kept at 30% for the first 2 min (0–2 min), then reached 100% in 18 min (20 min) and held for 20 min (40 min), linearly changed to 30% in 0.01 min (40.01 min), and finally maintained for 4.99 min (45 min). The flow rate was 0.40 mL/min and the column temperature was set at 45°C.

Mass spectrometry was performed using a Thermo Scientific Orbitrap Elite mass spectrometer with a heated electrospray ion source (HESI-II) (Thermo Scientific, United States), and the eluents were monitored in both positive and negative modes. Briefly, for the positive mode, the parameters were as follows: heater temperature 300°C; sheath gas flow rate 45 arb; Aux gas flow rate 15 arb; sweep gas flow rate 1 arb; spray voltage 3.0 kV; capillary temperature 350°C; S-lens RF level 30%; and scan ranges 200–1500. For the negative mode, the parameters were as follows: heater temperature 300°C; sheath gas flow rate 45 arb; Aux gas flow rate 15 arb; sweep gas flow rate 1 arb; spray voltage 2.5 kV; capillary temperature 350°C; S-lens RF level 60%; and scan ranges 200–1500.

### LC–MS Date Processing and Lipid Identification

The raw data obtained from LC–MS analysis of all samples were processed initially by using Lipid Search v4.0.20 software (Thermo Scientific, United States). The data from each sample were then normalized to total area, and all data about variates [including retention time (rt) and charge-to-mass ratio (*m*/*z*)], sample number, and normalized peak intensities were imported into SIMCA-P^+^ 12.0 software (Umetrics, Umea, Sweden), where multivariate analyses including principal component analysis (PCA) and orthogonal partial least squares discrimination analysis (OPLS-DA) were performed to classify lipid samples. In addition, the OPLS-DA models were validated using a permutation test with 200 as the permutation number (Su et al., 2013).

The lipid metabolite information, including lipid types, number of saturated bonds, and differences in chain length of lipid molecules, were obtained by qualitative analysis using Lipid Search software (Thermo Scientific, United States). To select potential lipid biomarkers, the variable importance in the projection (VIP, VIP > 1) values of lipid metabolites in the OPLS-DA model and *P-*values (*P* < 0.05) acquired from the *t*-test analysis were regarded as the screening condition. In addition, fold change (FC) analysis was also conducted.

### Statistical Analysis

The growth performance, relative abundance (%) of lipids, and position distribution of fatty acids [including 16:0 (palmitic acid), 18:1*n*−9 (oleic acid), 18:2*n*−6 (linoleic acid), 18:3*n*−3 (linolenic acid), 20:4*n*−6 (arachidonic acid), 20:5*n*−3 (eicosapentaenoic acid), and 22:6*n*−3 (docosahexaenoic acid)] were assessed by using Student’s *t*-tests in SPSS 17.0 for Windows (SPSS Inc., New York, NY, United States) (Liu et al., 2019). The data were expressed as the mean ± SEM, and the level of significant difference was set at *P* < 0.05.

## Results

**FIGURE 1:**
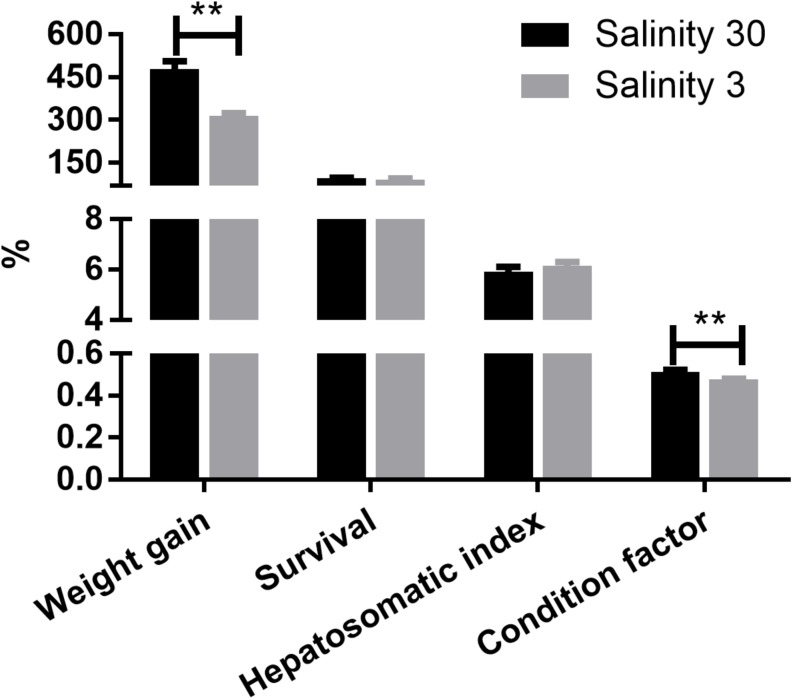
The weight gain (%), survival (%), hepatosomatic index (%), and condition factor (%) of *L. vannamei* at 30 and 3‰ salinity. Data are presented as the mean ± SEM (*n* = 4). Two asterisks (^∗∗^) indicate a highly significant difference (*P* < 0.01) between two salinities.

Weight gain and condition factor (*P* < 0.01) of the shrimp grown at 3‰ were significantly lower than those grown at 30‰. However, there were no significant differences in shrimp survival rate and hepatosomatic index between the two salinity groups (Figure 1).

**FIGURE 2:**
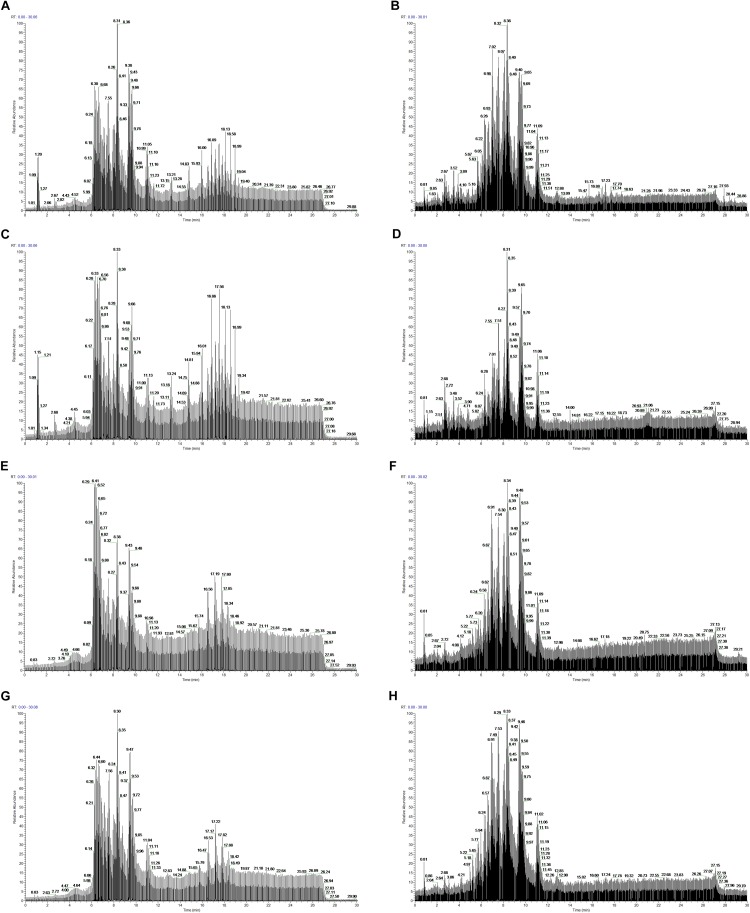
Typical total ion chromatograms from gill samples at 30 (**A**, ESI^+^ mode; **B**, ESI^–^ mode) and 3‰ salinity (**C**, ESI^+^ mode; **D**, ESI^–^ mode) and muscle samples at 30 (**E**, ESI^+^ mode; **F**, ESI^–^ mode) and 3‰ salinity (**G**, ESI^+^ mode; **H**, ESI^–^ mode).

**FIGURE 3:**
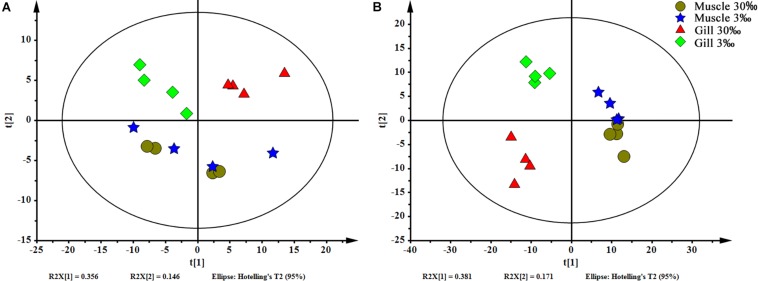
PCA scores plot of gills and muscles at two salinities. **(A)** ESI^+^ and **(B)** ESI^–^. Circle (brown), muscles at 30‰ salinity; star (blue), muscles at 3‰ salinity; triangle (red), gills at 30‰ salinity; and rhombus (green), gills at 3‰ salinity.

**FIGURE 4:**
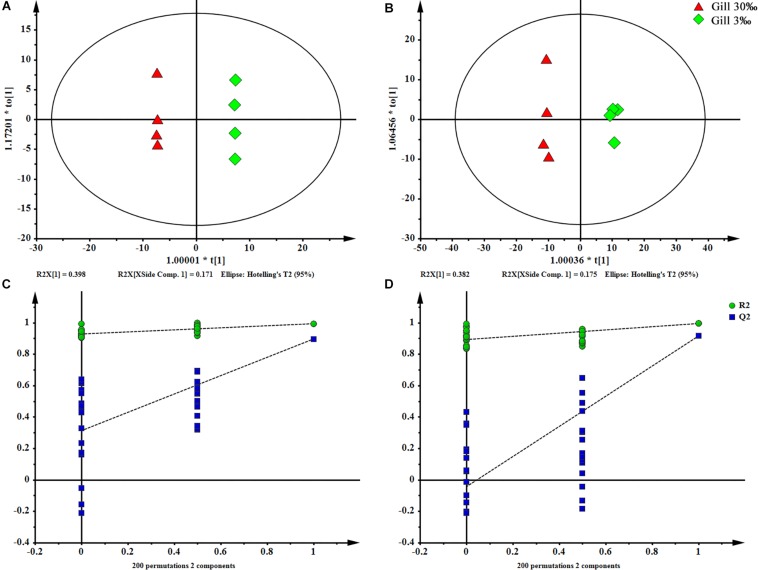
OPLS-DA scores plot (**A**: ESI^+^; **B**: ESI^–^) and permutation test plots with a 200 permutation number (**C**: ESI^+^; **D**: ESI^–^) of gills at two salinities. The permuted *Q*^2^ (blue) values located on the left side of the graph were lower than the original points to the right, indicating the validity of the OPLS-DA model.

**FIGURE 5:**
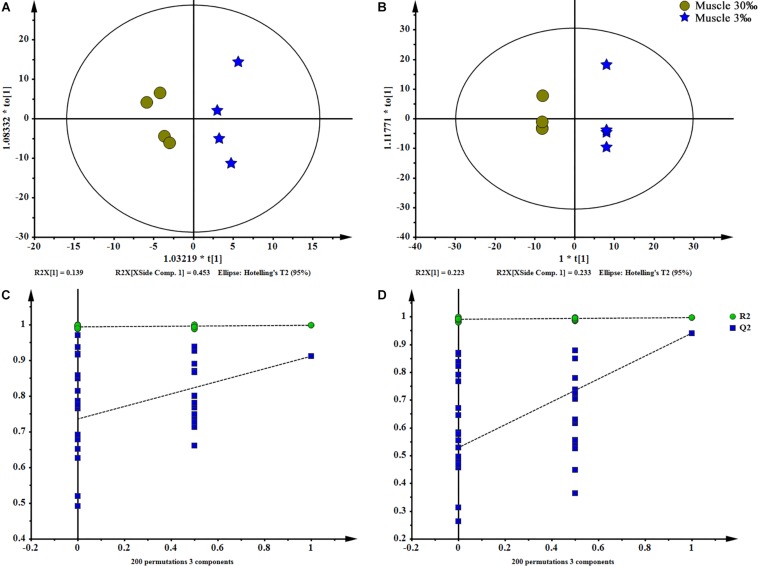
OPLS-DA scores plot (**A**: ESI^+^; **B**: ESI^–^) and permutation test plots with a 200 permutation number (**C**: ESI^+^; **D**: ESI^–^) of muscles at two salinities. The permuted *Q*^2^ (blue) values located on the left side of the graph were lower than the original points to the right, indicating the validity of the OPLS-DA model. The data obtained from muscles in ESI^+^ were not available.

In total, 404 metabolite peaks, including 93 peaks in positive mode and 311 peaks in negative mode, were selected for multivariate analysis (Figure 2). According to the values of the parameters for the PCA model (Table 1), all *R*^2^X (cum) were higher than 0.4, indicating the model was reliable. The PCA score plots obtained from four comparison groups showed four clusters in positive and negative ion scan modes by the first two components (Figure 3), respectively. Moreover, the two clusters corresponding to the gill samples in the score plots were separated more clearly in both ion scan modes by the first two components than those corresponding to muscle samples at the two salinities. The OPLS-DA score plots of gill groups (Figures 4A,B) and muscle groups (Figures 5A,B) showed clear separation. However, based on the *R*^2^Y values (0.937–1) and *Q*^2^ values (0.433–0.893) (Table 2), and the permutation test plots of positive and negative modes (gill group, Figures 4C,D; muscle group, Figures 5C,D), the data obtained from muscle groups in ESI^+^ were not available.

**TABLE 1:** Summary of the parameters in PCA model.

**Title**	**POS model**			**NEG model**		
		***R*^2^X**	***Q*^2^**		***R*^2^X**	***Q*^2^**
	***C***	**(cum)**	**(cum)**	***C***	**(cum)**	**(cum)**
All groups	2	0.502	0.266	3	0.673	0.400
G30‰ vs. G3‰	2	0.612	0.155	2	0.588	0.111
M30‰ vs. M3‰	2	0.641	0.279	2	0.550	−0.071

*The letters “G” and “M” are the abbreviations for gills and muscles, respectively. *C* is the number of components. *R*^2^X (cum) is the cumulative modeled variation in X-matrix. *Q*^2^ (cum) is the cumulative predicted variation in Y-matrix.*

**TABLE 2:** Summary of the parameters in OPLS-DA model.

**Title**	**POS model**				**NEG model**			
	***C***	***R*^2^X (cum)**	***R*^2^Y (cum)**	***Q*^2^** **(cum)**	***C***	***R*^2^X (cum)**	***R*^2^Y (cum)**	***Q*^2^** **(cum)**
G30‰ vs. G3‰	1 + 2	0.659	1	0.863	1 + 1	0.557	0.995	0.893
M30‰ vs. M3‰	1 + 1	0.592	0.937	0.433	1 + 4	0.835	1	0.886

*The letters “G” and “M” are the abbreviations for gills and muscles, respectively. *C* is the number of components, the numbers before and after the plus sign are the principal component and orthogonal components, respectively. *R*^2^X (cum) is the cumulative modeled variation in the X-matrix. *R*^2^Y (cum) is the cumulative modeled variation in the Y-matrix. *Q*^2^ (cum) is the cumulative predicted variation in the Y-matrix.*

**FIGURE 6:**
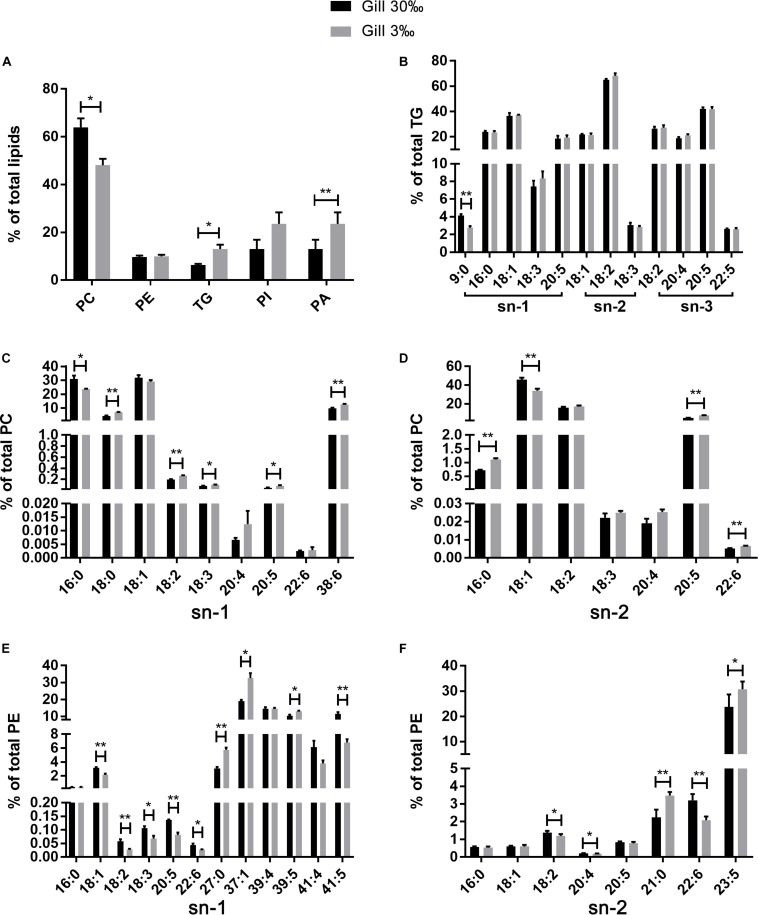
The relative abundance of specific lipids in total lipids **(A)** and the positional distribution of individual fatty acids in total TG **(B)**, total PC (**C**: sn-1 position; **D**: sn-2 position), and total PE (**E**: sn-1 position; **F**: sn-2 position) in gills of *L. vannamei*. Values are means ± SEM (*n* = 4). One asterisk (^∗^) and two asterisks (^∗∗^) indicate significant differences (*P* < 0.05) and highly significant differences (*P* < 0.01) between two salinities, respectively.

**FIGURE 7:**
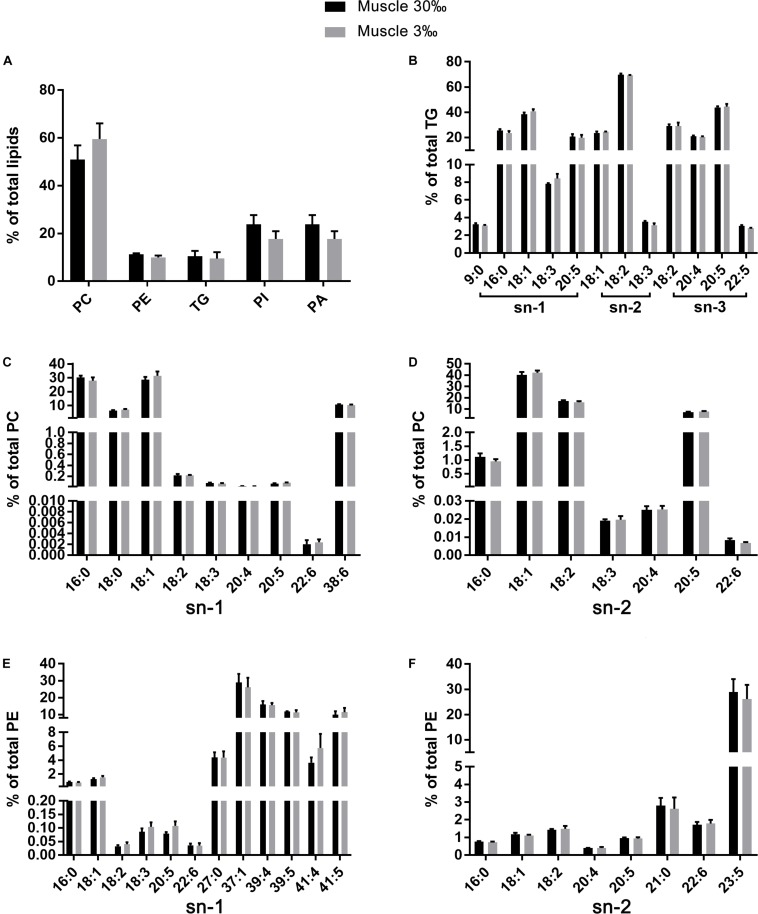
The relative abundance of specific lipids in total lipids **(A)** and the positional distribution of individual fatty acids in total TG **(B)**, total PC (**C**: sn-1 position; **D**: sn-2 position), and total PE (**E**: sn-1 position; **F**: sn-2 position) in muscles of *L. vannamei*. Values are means ± SEM (*n* = 4). One asterisk (^∗^) and two asterisks (^∗∗^) indicate significant differences (*P* < 0.05) and highly significant differences (*P* < 0.01) between two salinities, respectively.

A total of 37 significantly different lipid metabolites belonged to 6 lipid classes were then identified in muscle comparison groups, and 159 in gill comparison groups were classified into 11 lipids classes (Table 3). Among all lipid classes, phosphatidylcholine (PC), phosphatidylinositol (PI), phosphatidic acid (PA), phosphatidylethanolamine (PE), and triglyceride (TG) were the main lipid classes in both gills and muscles, regardless of salinities (Figures 6A, 7A). In detailed, the percentage of PC significantly reduced, but TG and PA significantly increased in gills of shrimp grown at 3‰. No lipid classes in muscle were affected by low salinity. Although PI was not significantly different between two salinity groups, regardless of muscle or gill, it showed opposite trends in both tissues; that is, it increased in the gill but decreased in the muscle. The percentage of PC tended to be higher in muscle tissue of shrimp grown at 3‰ compared to those grown at 30‰ salinity.

**TABLE 3:** Differential lipid metabolites in muscles and gills of *L. vannamei*.

**Lipid class**	**Muscle**			**Gill**			**Total**
	**Positive**	**Negative**		**Positive**	**Negative**		
	**ion mode**	**ion mode**	**Total**	**ion mode**	**ion mode**	**Total**	
Phosphatidylcholine (PC)	–	17	17	11	48	59	76
Phosphatidylethanolamine (PE)	–	9	9	9	36	45	54
Phosphatidylglycerol (PG)	–	–	–	8	–	8	8
Phosphatidic acid (PA)	–	2	2	–	6	6	8
Sphingomyelin (SM)	–	6	6	2	6	8	14
Lyso-phosphatidylcholine (LPC)	–	2	2	–	5	5	7
Lyso-phosphatidylethanolamine (LPE)	–	–	–	–	1	1	1
Phosphatidylinositol (PI)	–	–	–	2	–	2	2
Triglyceride (TG)	–	–	–	10	–	10	10
Diacylglycerol (DG)	–	–	–	2	–	2	2
Phosphatidylserine (PS)	–	1	1	–	13	13	14
Total	–	37	37	44	115	159	196

*“–” means fatty acid was not detected.*

The fatty acid compositions of each position of specific lipids were similar regardless of salinities or tissues. No differences were found in fatty acid compositions in all lipid classes in muscle of *L. vannamei* between the two salinity groups (Figures 7B–F). However, compared with shrimp at salinity 30‰, shrimp at 3‰ had significantly lower percentages 16:0 at the sn-1 position of PC, but higher percentage of 18:0, 18:2*n*−6, 18:3*n*−3, 20:5*n*−3, and 38:6 (Figure 6C). The percentage of 16:0, 20:5*n*−3, and 22:6*n*−3 except for 18:1*n*−9 was significantly higher in shrimp grown at 3‰ in the sn-2 position of PC (Figure 6D). At the sn-1 position of PE molecules, 18:1*n*−9, 18:2*n*−6, 18:3*n*−3, 20:5*n*−3, 22:6*n*−3, and 41:5 reduced significantly. Meanwhile, 27:0, 37:1, and 39:5 significantly increased (Figure 6E). At the sn-2 position, the percentages of 18:2*n*−6, 20:4*n*−6, and 22:6*n*−3 decreased, but percentages of 21:0 and 23:5 increased (Figure 6F). In the TG class, only the percentage of 9:0 at the sn-1 position significantly decreased in gills of shrimp at 3‰ than at 30‰ (Figure 6B).

**FIGURE 8:**
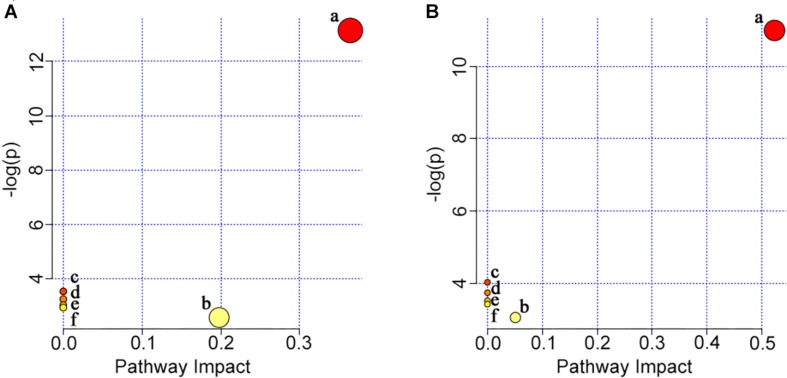
Summary of pathway analysis in gill **(A)** and muscle **(B)** with MetaboAnalyst 4.0. (a) Glycerophospholipid metabolism; (b) glycerolipid metabolism; (c) linoleic acid metabolism; (d) alpha-linolenic acid metabolism; (e) arachidonic acid metabolism; (f) glycosylphosphatidylinositol (GPI)-anchor biosynthesis.

The potential target metabolic pathway analysis based on the differential lipid metabolites obtained from gill and muscle samples (Supplementary Tables S1, S2, respectively) revealed that those metabolites were responsible for the metabolism of glycerophospholipid, linoleic acid, alpha-linolenic acid, arachidonic acid, glycerolipid, and for the biosynthesis of glycosylphosphatidylinositol (GPI)-anchor (impact-value ≥ 0.01, Figure 8).

## Discussion

In this study, *L. vannamei* cultured at 30‰ salinity exhibited better growth than those at 3‰ salinity. Similarly, growth of *L. vannamei* at 2 and 4‰ were significantly lower than that of shrimp at 30‰ (Laramore et al., 2001; Gao et al., 2016). As a euryhaline species, *L. vannamei* was reported to have an optimal salinity range of 20–25‰ for growth (Huang et al., 2004; Li et al., 2017). It is worth noting that 30‰ salinity is the actual salinity of local marine aquaculture. Our study suggested that shrimp had better growth performance at 30‰ salinity for being close to the hemolymph isotonic point, also further confirmed that ambient water at 3‰ was definitely stressful for *L. vannamei*.

In this study, the lipid components in gill tissues obtained from *L. vannamei* were very similar to those of muscle, regardless of ambient salinities, and phospholipids were the principal lipids. The phospholipid composition was found to be principally formed of relatively large amounts of PC, followed by PI and PA in the present study. As reported, phospholipids also proved to be the principal lipids in *Eriocheir sinensis*, but it was interesting to note that PC was the main phospholipid, and PE was the second most abundant phospholipid in both gill and muscle tissue (Chapelle, 1977), illustrating the differences in lipid content between the two crustacean species. Classes and composition of phospholipids present in different animals have been characterized, and each animal tissue has its own pattern of phospholipid classes (Ju et al., 2011). PC and PE were the two most important phospholipids in muscle tissue of Nile tilapia (Liu et al., 2019). Interestingly, PE was the dominant phospholipid in *Mya truncate* and in the muscle tissue of terrestrial animals (Bevers et al., 1999; Gillis and Ballantyne, 1999), indicating that composition of lipids is species-dependent.

According to the analysis of percentages, the relative abundances of PC significantly reduced, while TG and PA increased, in gills of shrimp grown at 3‰ salinity. PC acted as a choline “storage” molecule, and the choline could be metabolized to organic osmolytes such as betaine via glycine, serine, and threonine metabolic pathways (Athamena et al., 2011; Jiang et al., 2019). As reported, choline was reduced under hyperosmotic stress while elevated under hypo-osmotic stress in the gills of tongue sole (*Cynoglossus semilaevis*) (Jiang et al., 2019). The osmolality of hemolymph of crustaceans decreased after acclimation to dilute seawater (Lucu et al., 2008). Kettunen et al. (2001) demonstrated that betaine in the duodenal epithelium of broiler chicks played a crucial role to maintain water balance, thereby improving the osmoregulatory ability. Therefore, the reduction of PC in gill tissue of shrimp grown at 3‰ may be related to choline elevation under hypo-osmotic stress, regulation of the water permeability of gills, and maintenance of the normal morphology and function of cells.

It is worth to note that gill PI showed an increased trend in shrimp grown at 3‰, which was regarded as the phospholipid the least implicated in acclimation mechanisms in crabs (Chapelle, 1986). Furthermore, saturated fatty acids (SFAs) constituted the prevalent class of fatty acids in phospholipid PI detected in gill samples, including 17:0 and 34:0. Similar results were found in *Serripes groenlandicus* and *M. truncata* due to the high levels of 16:0 in PI of these two Arctic marine bivalve mollusks (Gillis and Ballantyne, 1999). At present, it is difficult to explain the exact functions of these specific fatty acids, 17:0 and 34:0. Perhaps both SFA and simpler fatty acids composition could help to improve the stability of PI function, suggesting PI plays highly specific roles in bio-membrane function. In cells, PI was used as a substrate in the plasma membrane, and PI cycle was the major metabolic pathway for PI biosynthesis, linked to actin polymerization and able to change membrane shape (Bozelli and Epand, 2019). Hence, we hypothesized that PI might be one of the important structural lipid components in membranes and had the capability to regulate bio-membrane structure or change cell shape, or could quickly fill the “vacancy” due to PC reduction.

The proportion of PA extremely significantly increased in gill of shrimp grown at 3‰ salinity. In the PI-cycle reaction, PI is transferred from the endoplasmic reticulum to the plasma membrane in exchange for PA moving in the opposite direction (Bozelli and Epand, 2019). Part of PA is synthesized in the endoplasmic reticulum and transported to the mitochondrial outer membrane. Moreover, PA has been reported to inhibit mitochondrial division and to stimulate mitochondrial outer membrane fusion, preventing the creation of too many mitochondria by excess division that might potentially compromise oxidative phosphorylation (Kameoka et al., 2018). Hence, the increase in PA may be closely related to mitochondrial fission and fusion, which play critical roles in maintaining physiological functions of mitochondria when gill cells are under environmental stresses, enhancing the synthesis of ATP and providing sufficient energy for osmoregulation. Also as reported, PA was related to the enhancement of diphosphatidylglycerol (DPG) synthesis when *E. sinensis* was transferred from sea water to fresh water, and the rapid renewal of DPG was thought to directly enhance mitochondrial activity (Chapelle and Zwingelstein, 1984). The changes in number or activity of mitochondria organelles might occur in response to the increased energy requirement required by the active transport mechanism because mitochondria have the principal function to form ATP (Chapelle and Zwingelstein, 1984; Lucu et al., 2008). In contrast, the lack of mitochondrial division led to the enlargement of mitochondria, decreased the efficiency of mitochondrial transport into subcellular regions, and consumed more additional ATP compound (Youle and van der Bliek, 2012). Furthermore, it was reported that PA played a fundamental role in the biosynthesis and metabolism of TG (Stanley et al., 2013). To maintain the balance of PA content in the cells, a portion of the PA might be used to synthesize TG, further providing sufficient energy for the gills.

Osmoregulation is an energy-dependent process, and aquatic animals are forced to spend more additional energy for modulating and stimulating ion transport mechanisms when challenged with salinity stress (Tseng and Hwang, 2008; Li et al., 2017). As good indicators for assessing the energy utilization, oxygen consumption and respiratory quotients of shrimp at 3‰ were significantly higher than those of shrimp at 17 and 32‰ salinity (Li et al., 2007). Similarly, higher oxygen consumption was needed in juvenile pompanos reared at 3‰ salinity than for those cultured in the other experimental salinities (6, 12, and 32‰) (Abou Anni et al., 2016). TG is a major class of neutral lipid used for energy storage (Liu et al., 2019), and the increase in TG synthesis was expected. The present research also indicates that shrimp grown at salinity of 3‰ require more energy than at 30%.

Both the total content and positional distribution of fatty acids in lipid molecules affected the nutritional values of the lipids and their utilization in metabolism (Karupaiah and Sundram, 2007; Liu et al., 2019). In this study, the composition and relative abundance of fatty acids at the same position of the specific lipid class was similar in *L. vannamei* regardless of ambient salinities or tissues, indicating that distribution of fatty acids in lipids was far from random, and the utilization of lipid classes is highly specific in *L. vannamei*. Fatty acids, such as 20:5*n*−3 and 22:6*n*−3, showed significantly positive genetic correlations, suggesting that the high values have potential for genetic improvement (Nolasco-Alzaga et al., 2018). However, other fatty acids like 20:4*n*−6 showed an exceptionally low level of heritability, indicating a larger role for environmental regulation than genetic. As reported, the lipid molecules and fatty acid contents could also be modified by different dietary oils and different salinities (Chapelle et al., 1982; Chen et al., 2014; Chen et al., 2015; Liu et al., 2019). Thus, work on this topic should be further conducted.

In this study, a variety of unsaturated fatty acids were found in PE, which was regarded as the most highly unsaturated phospholipid in a marine animal, playing an important role in the permeability of biological membranes via regulation of fluidity (Chapelle et al., 1982). Seven specific fatty acids including 20:4*n*−6 and 20:5*n*−3 had important nutritional value in organisms (Liu et al., 2019). As reported, 20:4*n*−6 and 20:5*n*−3 were abundant in the posterior gills of crabs (Chapelle and Zwingelstein, 1984), which were found mainly in TG in this study. Importantly, dietary supplementation with 20:5*n*−3 and 22:6*n*−3 could change gill water permeability of *L. vannamei* exposed to low salinities (Hurtado et al., 2007). Thus, we hypothesized that the role of 20:4*n*−6 and 20:5*n*−3 is related to energy supplying and changing water permeability of gills, and *L. vannamei* could rapidly respond to changes in environmental salinity, but more information should be obtained in the future to further confirm this.

## Conclusion

In conclusion, growth performance of *L. vannamei* would be inhibited when grown at salinity 3‰. Shrimp gill, as the organ directly connects with ambient water, is more sensitive than muscle because higher number of significantly changed lipids was found in shrimp gill. The significantly changed lipids were related to bio-membrane structure, mitochondrial function, fatty acid utilization, energy supply, or organic osmolyte content, for improving shrimp osmoregulatory capacity. The dramatic change in lipid profile is a significant physiological strategy that *L. vannamei* employs to cope with low salinity stress.

## Data Availability

All datasets for this study are included in the manuscript and the Supplementary Files.

## Ethics Statement

All experimental procedures were conducted in conformity with institutional guidelines for the care and use of laboratory animals in Hainan University, Haikou, China.

## Author Contributions

EL and QZ conceived and designed the research. MH, JX, and CX conducted the research. YD and MH finished the cultured experiment. YD, MH, YZ, and QC performed the statistical analysis. MH and EL wrote the manuscript. All authors contributed to the manuscript revision and approved the submitted version of the manuscript.

## Conflict of Interest Statement

The authors declare that the research was conducted in the absence of any commercial or financial relationships that could be construed as a potential conflict of interest.

## Supplementary Material

TABLE S1 The raw data of differential lipid metabolites using for pathway analysis in gill. VIP > 1 and *P-*values < 0.05 were regarded as the screening condition. *m*/*z* is the mass-to-charge ratio of metabolites. FA is the abbreviation of fatty acid and VIP is the abbreviation of variable importance in the projection.

TABLE S2 The raw data of differential lipid metabolites using for pathway analysis in muscle. VIP > 1 and *P-*values < 0.05 were regarded as the screening condition. *m*/*z* is the mass-to-charge ratio of metabolites. FA is the abbreviation of fatty acid and VIP is the abbreviation of variable importance in the projection.
